# Respiratory Epithelial Orbital Cyst: A Case Report and Literature Review

**DOI:** 10.1155/2018/7256871

**Published:** 2018-02-14

**Authors:** Sally Al Abdulmohsen, Ayman Ayoubi, Sadeq Al-Dandan

**Affiliations:** ^1^Department of Anatomical Pathology, King Fahad Medical City, Riyadh, Saudi Arabia; ^2^Department of Ophthalmology, King Fahad Medical City, Riyadh, Saudi Arabia

## Abstract

A 44-year-old male with schizophrenia presented with progressive right proptosis for one year and conjunctivitis for two months. An orbital cyst was seen in the superotemporal region on computerized tomography and was surgically removed. There was no history or radiological signs of paranasal sinus disease or previous trauma. Histopathologic evaluation revealed a cyst lined with respiratory epithelium. Respiratory choristomatous cysts of the orbit are considered rare in both pediatric and adult patients. We review the literature of respiratory orbital cysts and conclude that they tend to present in adults and should be considered in the differential diagnoses of orbital cysts.

## 1. Introduction

A choristoma is defined as normal tissue present in an abnormal location. The most common choristomatous orbital cysts are dermoid followed by epidermoid cysts in both adult and pediatric populations. Except for mucoceles, which are acquired due to chronic sinusitis, choristomatous cysts are generally more prevalent in the pediatric age group. However, the uncommon, respiratory epithelial orbital cysts tend to present in adults and may be underrepresented in literature.

## 2. Our Case

**Figure 1:**
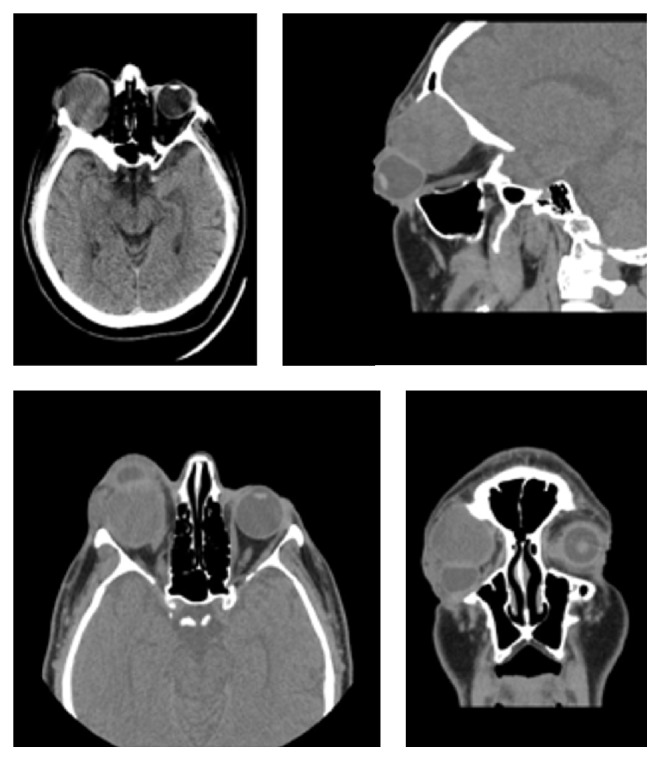
CT axial, sagittal, and coronal views of cyst causing proptosis.

A forty-four-year-old man with schizophrenia presented with right proptosis for one year and a history of pain for two months. The patient was uncooperative on physical examination. There were severe proptosis, poor light perception in the affected eye with a complete frozen globe, and total corneal opacity, associated with prominent eye congestion and eyelid swelling. The orbital mass was palpated above the eyelid. Computed topography scan (Figure 1) showed an extraconal intraorbital tumor in the upper outer quadrant of the right orbit with orbital roof bony erosion, measuring 41 × 39 × 37 mm. The mass displaces the superior rectus muscle as well as the lacrimal gland, causing marked proptosis. No intracranial involvement was seen. The paranasal sinuses were well aerated and clear. The radiological differential diagnoses included epidermoid cyst and adenoid cystic carcinoma.

**Figure 2:**
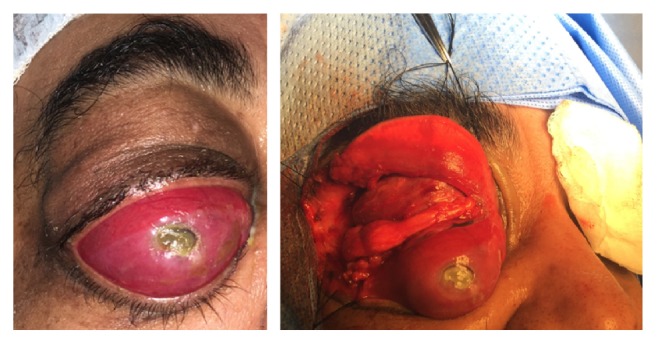
Anterior view with total corneal opacity.

**Figure 3:**
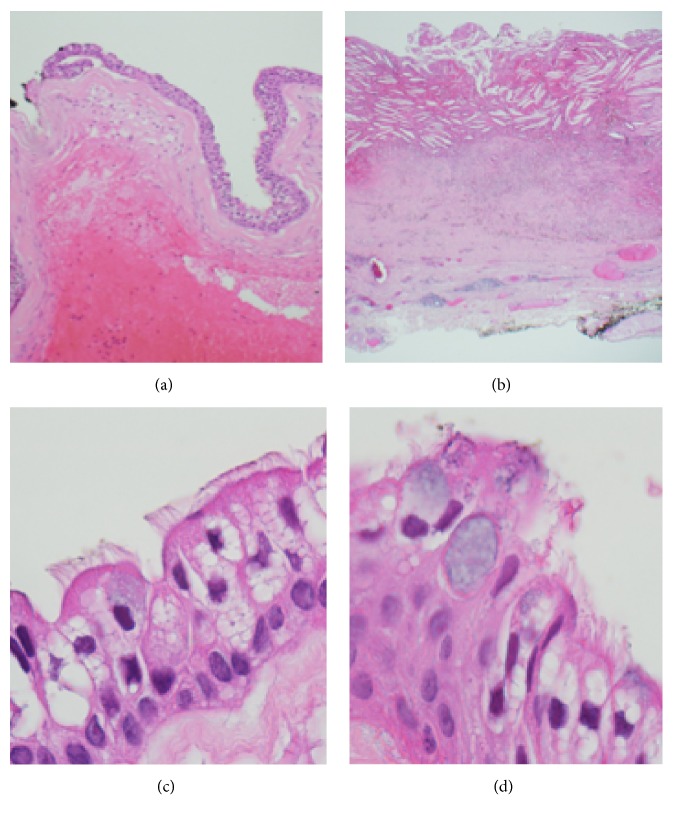
(a) shows the pseudostratified ciliated columnar epithelium with a (b) xanthogranulomatous reaction. (c) Cilia and (d) goblet cells are demonstrated.

Anterior orbitotomy through transconjunctival approach (Figure 2) was then performed. Bony destruction at the superior orbital rim was seen. The cyst was excised, and the patient was given postoperative antibiotics (fortified gentamycin and fortified cephazolin). The cyst was sent to pathology in formalin having been previously opened and was submitted entirely for histopathological evaluation. The cyst was found to be lined with benign pseudostratified ciliated columnar epithelium with goblet cells (respiratory epithelium) and an associated xanthogranulomatous reaction (Figure 3). Complete ptosis of the affected eye was seen postoperatively. The patient was then lost to follow-up.

## 3. Discussion

Choristomas are believed to occur during development. The most common choristomas of the orbit are dermoid and epidermoid cysts. Other orbital choristomas include those arising from lacrimal, adipose, cartilage, bone, and brain [1] as well as enterogenous [2] rests. Acquired orbital respiratory cysts are caused by extension of paranasal sinus mucosa either by herniation because of trauma or by pressure erosion of the thin intervening bone by an expanding mucocele [3]. Mucoceles arise from prolonged obstruction to outflow and they communicate with parent paranasal sinuses [4]. Respiratory epithelial cysts with no connection to paranasal sinuses and not associated with sinus disease or trauma are believed to be rare.

Reported examples of respiratory epithelial cysts caused by trauma include ones arising six months after an orbital fracture repair [5]. One paper reported 10 cases of post-traumatic respiratory epithelial cyst with a mean presentation of 17.4 years after the original trauma [6]. In the case of our patient with schizophrenia, a reliable history cannot be obtained to entirely exclude trauma. Nevertheless, the superotemporal region is the most common site of choristomatous cysts of the orbit, followed by the superomedial and, to a lesser extent, the inferior orbital region [7]. Therefore, the absence of radiological signs of past trauma and the superotemporal location both support the diagnosis of a choristomatous cyst in our patient. The late presentation may be the result of gradual enlargement and does not indicate that it is acquired.

Dermoid cysts account for over 40% of all orbital lesions of childhood and for 89% of all orbital cystic lesions of childhood that need biopsy or surgical removal [8]. All the commoner cystic lesions of the orbit apart from mucoceles present most commonly in patients aged 18 or less [9]. However, choristomatous respiratory epithelial cysts, unlike the common dermoid and epidermoid cysts, tend to present later in life with a gradually progressive history over an extended period [3]. We note that this is not the rule and that there are reported cases of respiratory epithelial cysts of the orbit in a three-year-old [10] and a nine-year-old [11], which were surgically removed to relieve symptoms. The nasal cavity and sinuses are lined with pseudostratified ciliated columnar epithelium with goblet cells derived from the endoderm during intrauterine development of the maxillary or sphenoid sinuses in the middle trimester [3]. Dermoid and epidermoid cysts are lined with keratinizing stratified squamous epithelium with the former including skin appendages. The less common, conjunctival cyst is lined with nonkeratinizing stratified squamous epithelium with goblet cells. Lacrimal ducts/canaliculi epithelium is of the nonkeratinizing squamous types while the nasolacrimal sac and duct are lined with stratified columnar epithelium with goblet cells that is never ciliated. Therefore, the presence of ciliated columnar epithelium in the orbital region indicates its choristomatous nature.

Generally, the diagnosis of primary respiratory choristomatous epithelial cyst of the orbit should be considered if it is located temporally, if there is no history of prior trauma or surgery, and, finally, if the symptoms present over a long period [12]. Ten cases of choristomatous respiratory orbital cysts found in English literature are shown (Table 1), demonstrating the medial, upper eyelid as well as the common lateral location at presentation. The table also delineates a wide age range of 3–88 years and highlights the higher incidence in adults, particularly females.

## 4. Conclusion

Acquired respiratory cysts of the orbit should be included in the differential diagnoses of orbital cysts in adults with paranasal sinus disease or history of trauma or surgery. Extension of a mucocele in the case of chronic paranasal sinusitis or herniation of paranasal epithelium due to trauma is implicated in the pathogenesis of acquired respiratory cysts. Primary respiratory choristomatous cysts of the orbit, on the other hand, are developmental rests that commonly occur in the superotemporal region, tend to present in adults, and may be more common than is represented in literature.

## Figures and Tables

**Table 1:** Primary choristomatous respiratory orbital cysts.

Case report	Age	Gender	Site
1 [10]	3	F	Lateral
2 [11]	9	F	Inferior medial
3 [13]	23	F	Superior lateral
4 [14]	24	F	Superior medial
5 [4]	26	F	Upper eyelid
	38	M	Superior lateral
6 [12]	37	M	Lateral
	35	F	Superior lateral
7 [15]	79	M	Upper eyelid
8 [16]	88	F	Superior medial
